# Novel TetR family transcriptional factor regulates expression of multiple transport-related genes and affects rifampicin resistance in *Mycobacterium smegmatis*

**DOI:** 10.1038/srep27489

**Published:** 2016-06-07

**Authors:** Huicong Liu, Min Yang, Zheng-Guo He

**Affiliations:** 1 National Key Laboratory of Agricultural Microbiology, College of Life Science and Technology, Huazhong Agricultural University, Wuhan 430070, China

## Abstract

Transport-related genes significantly affect bacterial antibiotic resistance. However, the effects of these genes and their regulation of bacterial drug resistance in several mycobacterial species, including the fast-growing *Mycobacterium smegmatis*, the pathogen *M. tuberculosis* and *M. avium* have not been clearly characterized. We identified Ms4022 (MSMEG_4022) as a novel TetR family regulator that activates the expression of seven transport-related genes and affects drug resistance in *M. smegmatis*. Overexpression of Ms4022 inhibited *M. smegmatis* growth and enhanced mycobacterial resistance to the anti-tuberculosis drug rifampicin (RIF). By contrast, the Ms4022-deleted mycobacterial strain has shown sensitive to RIF. Ms4022 recognized three 19 bp non-palindromic motifs containing a 9 bp conserved region at their 5′ end and it directly regulated seven transport-related genes, which affects mycobacterial resistance to RIF. Overexpression of three of seven transport-related genes (Ms1448, Ms1613, and Ms5278) inhibited the growth of *M. smegmatis*. This study improves our understanding of the function of mycobacterial transport-related genes and their regulation of bacterial drug resistance.

The occurrence and spread of bacterial drug resistance, especially multi-drug resistance (MDR), in several important human pathogens (e.g., *Mycobacterium tuberculosis*) pose a serious threat to human health worldwide. Recent studies have indicated that the existence of drug efflux systems, which can pump drugs out of bacterial cells and result in bacterial resistance to drugs, is one of the important causes of bacterial antibiotic resistance^1^,^2^. However, the regulatory mechanisms of bacterial drug resistance involving drug efflux remain unclear.

*M. tuberculosis* (MTB), the causative agent of tuberculosis (TB), continues to be a major global health problem^3^. Several mycobacterial species have different efflux pump genes associated with resistance to multiple drugs^4^,^5^,^6^. For example, LfrA is a multidrug efflux pump and activates the multidrug efflux in *M. smegmatis*^7^. The *iniBAC* operon encodes a typical efflux pump and could function as an MDR pump system in *M. smegmatis* and *M. tuberculosis*^8^. Multidrug-resistant and extensively drug-resistant *M. tuberculosis* strains frequently exhibit increased expression of this efflux system^9^. Many drug transporter regulatory proteins, including activators and repressors, have been identified in recent years. TetR family transcription factors regulate diverse physiological functions in bacteria. They control physiological processes, such as catabolic pathways, biosynthesis of antibiotics, and osmotic stress, in the pathogenicity of gram-negative and gram-positive bacteria^10^. The members of this family are often employed as negative regulators that inhibit the expression of target genes. For example, EmrR in *Escherichia coli* and QacR in *Staphylococcus aureus* negatively regulate MDR pumps^11^,^12^. EthR is a repressor of the TetR/CamE family associated with ethionamide resistance in *M. tuberculosis*^13^.

*M. smegmatis,* a fast-growing nonpathogenic mycobacterium, has been widely utilized as a model organism for the study of the mechanisms of gene regulation in extremely slow-growing mycobacterial species, such as *M. tuberculosis*^14^,^15^. Interestingly, *M. smegmatis* is also a suitable model for the study of the regulatory mechanism of mycobacterial drug resistance^16^. In particular, more than 500 potential regulatory factors and 600 transport-related genes are encoded by the genome of *M. smegmatis* (GenBank accession number CP000480). However, the physiological roles of these regulators and transport-related genes and their relationships with bacterial drug resistance remain unknown.

In this study, we characterize a new TetR family transcriptional factor, Ms4022, as a positive regulator in *M. smegmatis*. Ms4022 activates the expression of seven transport-related genes and affects the RIF resistance and growth of *M. smegmatis*. This study enhances our understanding of transport-related gene regulation and drug resistance in mycobacteria.

## Materials and Methods

### Strains, enzymes, plasmid, regents and DNA primers

*E.coli* DH5α cells were used to construct the recombinant plasmids. *E.coli* BL21 cells (DE3) and pET28a purchased from Stratagene (La Jolla, CA, USA) were used to express *M. smegmatis* protein. Restriction enzymes, T4 ligase, dNTPs and all antibiotics were purchased from TaKaRa Biotech (Shiga, Japan). All primers were synthesized by Tsingke Biological Technology (Wuhan, China) (Supplementary Table 1 and 2). DNA purification kits were purchased from Waston Biotechnologies (Wuhan China). All plasmids used in this study were listed in Supplementary Table 3. Antisera were purchased from the Laboratory Animal Centre, Institute of Virology, Chinese Academy of Sciences, Wuhan, China.

### The screening of rifampicin (RIF) related transcriptional regulators

Over 500 transcriptional regulator genes were amplified from *M. smegmatis* genomic DNA. The gene fragments were mixed as a pool and cloned into pMV261 vector to construct the regulatory genes overexpression plasmids library. The plasmids library were electrophoretic transferred into *M. smegmatis* mc^2^ 155, and the strains were screened on 7H10 plates containing 1.5 μg/mL RIF. As a result, those having increased RIF resistance or decreased RIF susceptibilities were identified as primary candidates. To avoid random mutations that may contribute to RIF resistance, plasmid were extracted from each of the primary candidates, and transformed into the wild type *M. smegmatis* and assayed thrice in a similar way. In final, the increased RIF resistance is sufficient to attribute to the overexpression of the corresponding transcriptional regulator.

### Electrophoretic mobility shift assay

Electrophoretic mobility shift assay (EMSA) was used to detect the DNA binding ability of Ms4022. DNA fragments for the DNA binding activity assays were from *M. smegmatis* genomic DNA or synthesized directly by Tsingke Biological Technology (Wuhan, China). The reaction (20 μl) for EMSA contained DNA and different concentrations of Ms4022 and containing 50 mM Tris–HCl (pH 7.5), 10 mM MgCl_2_, and 50 mM NaCl. The DNA and reaction mixtures were incubated at 4 °C for 30 min with various amounts of Ms4022, then subjected to 5% native PAGE using 0.5× Tris/borate/EDTA (TBE) as running buffer. Electrophoresis was performed at 150 V at room temperature for 2 hrs. Images were acquired by Typhoon Scanner (GE Healthcare, Little Chalfont, Buckinghamshire, UK).

### Footprinting assay

The *Ms4022* promoter DNA (upstream 500 bp of *Ms4022*) was amplified with reverse primer labeled with Fluorescein Isothiocyanate (FITC). The purified DNA fragment was added to the reaction mixture (containing various amounts of Ms4022) at 4 °C for 30 min as in EMSA. All of the mixtures were treated with DNaseI (1 unit, Fermentas) (Fermentas China Co., Ltd, Shenzhen China) at 37 °C for 2 min 30 s as described previously^14^. The results were analyzed with Applied Biosystems 37030XL DNA analyzer (manufactured by Tsingke Company, Wuhan, China).

### Chromatin immunoprecipitation Assay

Chromatin immunoprecipitation (ChIP) assay was used to detect the DNA binding ability of Ms4022 *in vivo*. Ms/wt and Ms/ΔMs4022 strains were cultivated in 100 ml 7H9 medium to log phase (OD_600_ ≈ 1.0). 1% formaldehyde was used to fix the cells for 30 min, then added 125 mM glycine for 5 min to stop the reaction, and then the cells were harvest. Cells were treated as described previously^15^. Samples of those were incubated with antibodies of Ms4022 or preimmune serum for 3 h at 4 °C. The complexes were treated as described previously^15^. The protocol of PCR amplification contained one denaturation step of 5 min at 95 °C, then 26 cycles of 1 min at 95 °C, 1 min at 60 °C and 1 min at 72 °C.All of the primers used here were list in supplementary Table 1.

### Quantitative real-time PCR

The *M. smegmatis* wild type strain (Ms/wt) and recombination strains (Ms/ΔMs4022, Ms/pMV261 and Ms/pMV261-Ms4022) were incubated in 7H9 medium to log phase (OD_600_ ≈ 1.0). Then cells were harvest and storage at −80 °C. RNA was extracted from these cells as described previously^15^, then digested with DNaseI and was used as template for synthesis of cDNA. Reactions were carried out in a Bio Rad CFX RT-PCR machine (Bio Rad, California, USA). Fold change in gene expression was calculated by the 2^−ΔΔCt^ method^17^.

### Determination of Mycobacterium growth curves and the MIC of drugs

All of the recombinant strains grown at 37 °C in 7H9 with or without kana to log phase, then diluted in 100 ml 7H9 with or without drugs to an OD_600 _of 0.1. Drug concentration is 8 μg/ml Isoniazid (INH), 1.5 μg/ml Rifampicin (RIF) or 1 μg/ml ethambutol (EMB) respectively. All of the strains were grown at 37 °C at 160 rpm for 3–4 d. These samples were taken by equal time and measured the OD_600_ as described previously^16^.

For minimal inhibitory concentration (MIC) determination using tube dilution methods as described previously^18^. Mid-log phase strains were diluted up to 0.1 ml culture containing 10^5 ^CFUs (colony forming unit) and RIF was two-fold diluted at a volume of 2.5 ml in 7H9 without Tween-80. All of the strains were grown at 37 °C at 160 rpm for 3–4 d. The minimum concentration that prevented pellet formation was identified as MIC.

### Analysis of β-galactosidase activity

Promoter -*LacZ* fusions plasmids were constructed base on pMV261 for analyzing the β-galactosidase activity experiments as described previously^19^. The multiple clone site of the plasmid was reformed by inserting a MCM stream and the same sense mutation was performed in the *Eco*RI site of *lacZ*. Then a pMV261-MCM-*LacZ* vector was constructed firstly. Promoter of Ms4022 target genes were cloned by *Eco*RI/*Xba*I into pMV261-MCM-*LacZ* (digested with *Eco*RI/*Xba*I). The reporter plasmids were electrophorated into Ms/wt and Ms/ΔMs4022 strains. All recombination strains were grown in 7H9 at 37 °C to an OD600 of 0.6~0.8. β-galactosidase measurements were performed as described previously^19^.

### Southern blotting

A pMind derived suicide plasmid containing the hygromycin resistance gene was used to construct *Ms4022* knockout vector. The vector was electrophorated into Ms/wt stain. The Ms/ΔMs4022 was selected on 7H10 (BD, USA) plate containing 100 μg/ml hygromycin. Ms4022 deleted strain was identified by southern bolt analysis. Ms/wt and Ms/ΔMs4022 genomic DNA were digested with *Apa*I. The 391bp DNA probe of the upstream of the Ms4022 was amplified using special primers (Supplementary Table 1).

## Results

### Ms4022 inhibits cell growth and positively regulates RIF resistance in *M. smegmatis*

**Figure 1:**
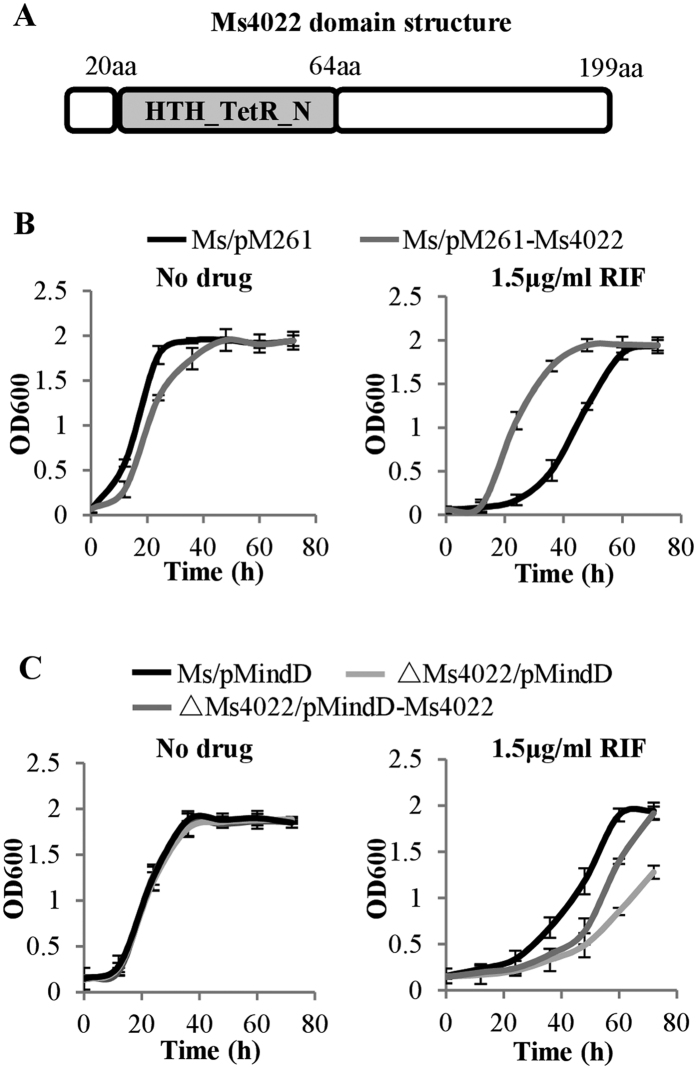
The effect of Ms4022 on mycobacterial growth in response to anti-tuberculosis drug rifampin (RIF). (**A**) Analysis of the structural characteristics of Ms4022. It contained a TetR_N superfamily domain within N domain. (**B**) Growth curves of the Ms/pMV261 and Ms/pMV261-Ms4022 were determined in the absence or presence of RIF. Ms/pMV261 and Ms/pMV261-Ms4022 were grown in 7H9 medium, which contains 30 μg/ml Kanamycin, without RIF (left panel) or 1.5 μg/ml RIF (right panel). (**C**) Ms/pMindD, ΔMs4022/pMindD and ΔMs4022/pMindD-Ms4022 were grown in 7H9 medium, which contains 30 μg/ml Kanamycin, with no drug (left panel) or 1.5 μg/ml RIF (right panel). For all these assays, aliquots were taken at the indicated times and the OD_600_ was measured. Each analysis was performed in triplicate, and standard errors were indicated as error bars.

Utilizing the transcriptional factor overexpression library of *M. smegmatis* constructed previously^20^, we screened the regulator-overexpressing strains on 7H10 plates containing 1.5 μg/mL RIF and identified a TetR family regulator in *M. smegmatis*, Ms4022 (Fig. 1A), which is associated with RIF resistance. As shown in Fig. 1B, the Ms4022-overexpressing strain (Ms/pMV261-Ms4022) grew slower than the control strain (Fig. 1B, left panel), and Ms/pMV261-Ms4022 showed increased resistance to RIF (Fig. 1B, right panel). Notably, no obvious effect was observed in the presence of INH or EMB (Supplementary Fig. 1). The Ms4022 deleted strain (ΔMs4022/pMindD) was more sensitive to RIF (Fig. 1C, right panel), while bacterial growth was unchanged when Ms4022 was deleted in *M. smegmatis* (Fig. 1C, left panel).The sensitivity of ΔMs4022/pMindD to RIF partially recovered when Ms4022 was complemented with pMindD plasmid (Fig. 1C, right panel). The MIC of Ms/ΔMs4022 (3.13 μg/ml) to RIF was two-fold lower than Ms/wt(6.25 μg/ml), whereas the MIC of Ms/pMV261-Ms4022 was four-fold higher than that of Ms/pMV261 (25 μg/ml). These results suggest that the TetR family regulator, Ms4022, contributes to the RIF resistance of *M. smegmatis*.

### Ms4022 recognizes three binding sites within the upstream sequence of its own operon

**Figure 2:**
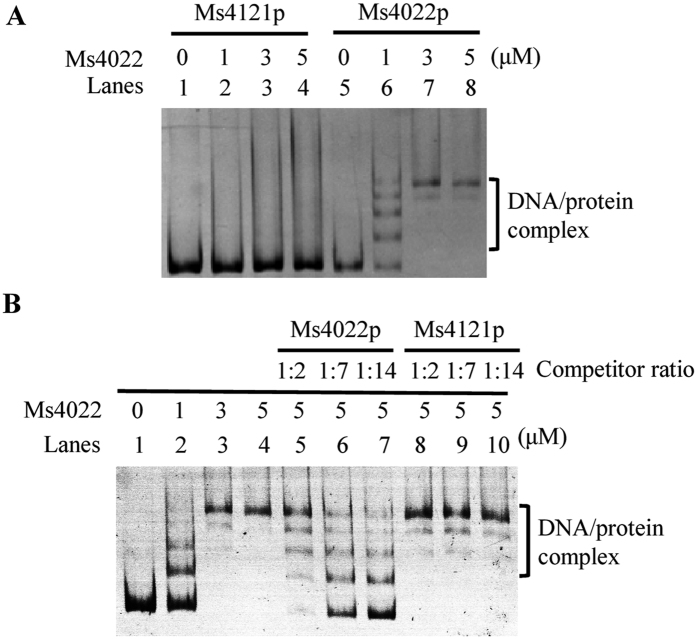
Specific binding of Ms4022 with its own promoter *in vitro*. (**A**) EMSA assays. Ms4022p or Ms4121p were co-incubated with various amount of Ms4022 protein as indicated. Ms4022 bound to Ms4022p, but not to Ms4121p, and form stable DNA/protein complexes. (**B**) EMAS assays for the specific binding of Ms4022 with Ms4022p. Unlabeled specific Ms4022 promoter and unspecific Ms4121 promoter were used to compete with the labeled Ms4022 promoter.

EMSA assay was utilized to detect the binding of Ms4022 to its upstream promoter DNA. As shown in Fig. 2A, the DNA/protein complex shifts significantly increased with increasing amounts of Ms4022 proteins (lanes 6 to 8). However, Ms4022 could not specifically bind to the control promoter Ms4121p (Fig. 2A). Furthermore, competition assay indicated that unlabeled Ms4022p, instead of Ms4121p, competitively inhibited the binding of Ms4022 to the labeled Ms4022 promoter DNA substrate (Fig. 2B). These results prove that Ms4022 specifically binds to Ms4022p.

**Figure 3:**
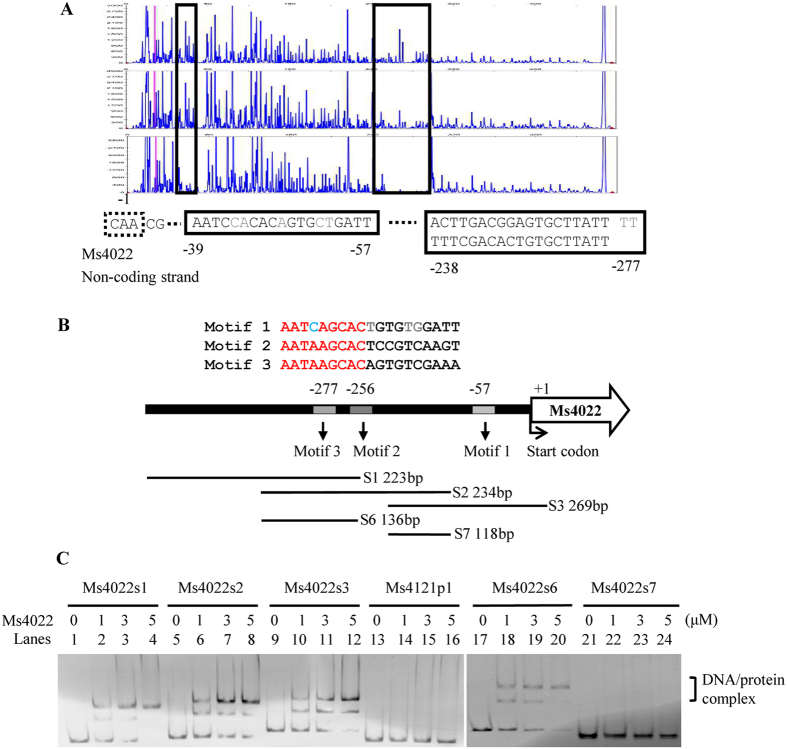
Identification of the DNA binding motif for Ms4022. (**A**) DNaseI footprinting assay. Ms4022p was incubated with increasing amounts of Ms4022 protein (from top to bottom), and digested by DNaseI. The protected region was shown with the box highlights. (**B**) Sequence and structural characteristics of the promoter region protected by Ms4022. The regions protected by Ms4022 were three 19 bp sequences. Their approximate positions were also given in this diagram. The translation start codon of Ms4022 was indicated in curved arrow. DNA fragments that used in the following EMSA assays were showed as well. Those fragments may contain binding motifs of Ms4022. (**C**) EMSA assay for DNA fragments of different truncated. The promoter of Ms4022 was truncated to different length to detect the DNA-binding region of Ms4022 on its own promoter. Different DNA fragments s1, s2, s3, s6, s7 and Ms4121p1 were incubated with various amounts of Ms4022 protein. Ms4121p1 was used as the negative control.

DNaseI footprinting assay was then employed to identify the binding motif of Ms4022. The non-coding strand of the DNA substrate was labeled with fluorescein isothiocyanate and then utilized as a substrate in the assay. When the mixture of Ms4022 and its promoter was co-incubated with DNaseI, two protected regions of Ms4022 promoter were detected (Fig. 3A). The first region AATCAGCACTGTGTGGATT extended from positions −57 to −39 (relative to the Ms4022 start codon). The second region AATAAGCACAGTGTCGAAA AA AATAAGCACTCCGTCAAGT extended from positions −277 to −238. Two defective palindromic motifs were separated by two nucleotides (AA). All three motifs had the similar sequence 5′ -AATCAGCAC -3′ in protected regions (Fig. 3B). To characterize the DNA binding motif for Ms4022 protein, DNA fragments in the Ms4022 promoter were amplified by PCR and then used as EMSA substrates. Unlike Ms4121p1, Ms4022 could bind to s1, s2, and s3 (Fig. 3B,C). Multiple binding sites were presumed to exist in the Ms4022 promoter. Different truncated DNA fragments within the Ms4022 promoter were amplified (s4 to s7). In the EMSA assay, Ms4022 bound to s4, s5, and s6 but not to s7 (Fig. 3C and Supplementary Fig. 3B). As previously mentioned, at least two motifs existed. One was in s2, and the other one was located near the translation initiation site of Ms4022.

**Figure 4:**
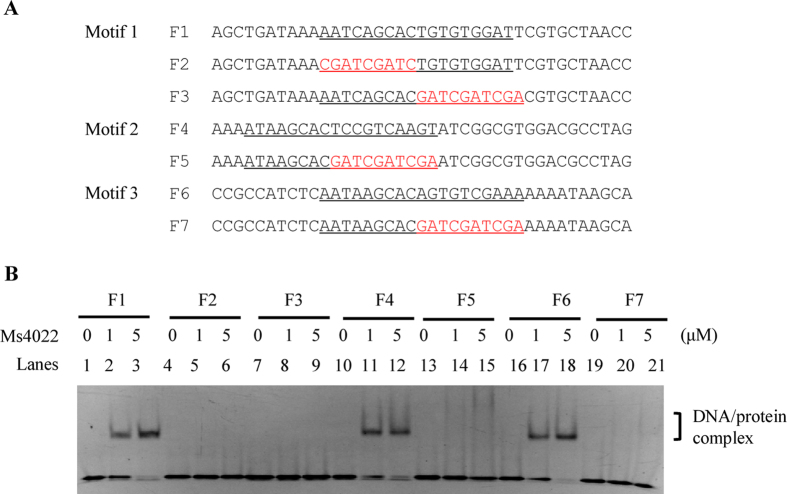
Specific binding of Ms4022 with the motifs. EMSA assays for the DNA-binding activity of Ms4022 on the DNA substrates with or without the complete binding motifs. Ms4022 could bind to F1, F4 and F6, but not F2, F3, F5 and F7 (with the mutant motif sequence).

To evaluate the effect of the motif sequence on Ms4022 recognition, we designed DNA substrates with and without motif mutation (Fig. 4A). The results show that when the conserved 9 bp regions were mutated, Ms4022 lost its binding ability to DNA fragments F2, F3, F5, and F7 (Fig. 4B).

These results indicate that three binding motifs existed in the promoter of Ms4022. The putative binding motifs for Ms4022 contained an imperfect palindromic sequence motif.

### Ms4022 binds with 22 promoters of transport-related genes

**Figure 5:**
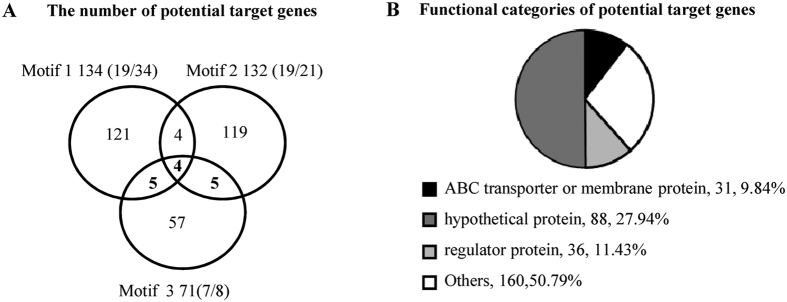
The number and functional categories of Ms4022 potential target genes in *M. smegmatis*. (**A**) Motif 1(AATCAGCACTGTGTGGATT), Motif 2 (AATAAGCACTCCGTCAAGT) and Motif 3 (AATAAGCACAGTGTCGAAA) were used to search the promoters of *M. smegmatis*. The number of Ms4022 potential target genes in *M. smegmatis* were shown in Venn figure. Some promoters of the target genes were detected by EMSA assays (see also supplementary Fig. 4). In those randomly selected promoters, binding rates for promoters were given within the brackets. (**B**) A function classification of the target genes in the context of COG categories.

A total of 315 potential target genes were identified by using these motifs to search the intergenic region of *M. smegmatis* separately. On the basis of the first, second, and third motifs, 134, 132, and 71 potential target genes were identified. Four promoters, namely, MSMEG_3427p, MSMEG_3799p, MSMEG_4022p, and MSMEG_5035p, had their promoter with the three binding motifs (Fig. 5A). Among them, promoters were randomly selected and applied to EMSA assays to confirm their binding with Ms4022. As a result, 19 of 34, 19 of 21, and 7 of 8 selected promoters bound with Ms4022 protein (Supplementary Fig. 4).

We analyzed the classification of the 315 potential genes by using the categories in the Cluster of Orthologous Groups (COG) of proteins. COG includes 31 membrane-associated transporter genes (Fig. 5B). By using the Web Logo tool^21^, a logo assay containing 19 bp non-palindrome sequence AANNAGCACNCCGTCGA NN was characterized, and the last two bases show very low similarity (Supplementary Fig. 5B). Among the 31 promoters of Ms4022 target genes, 22 promoters were bound by Ms4022 *in vitro* (Supplementary Fig. 4).

**Figure 6:**
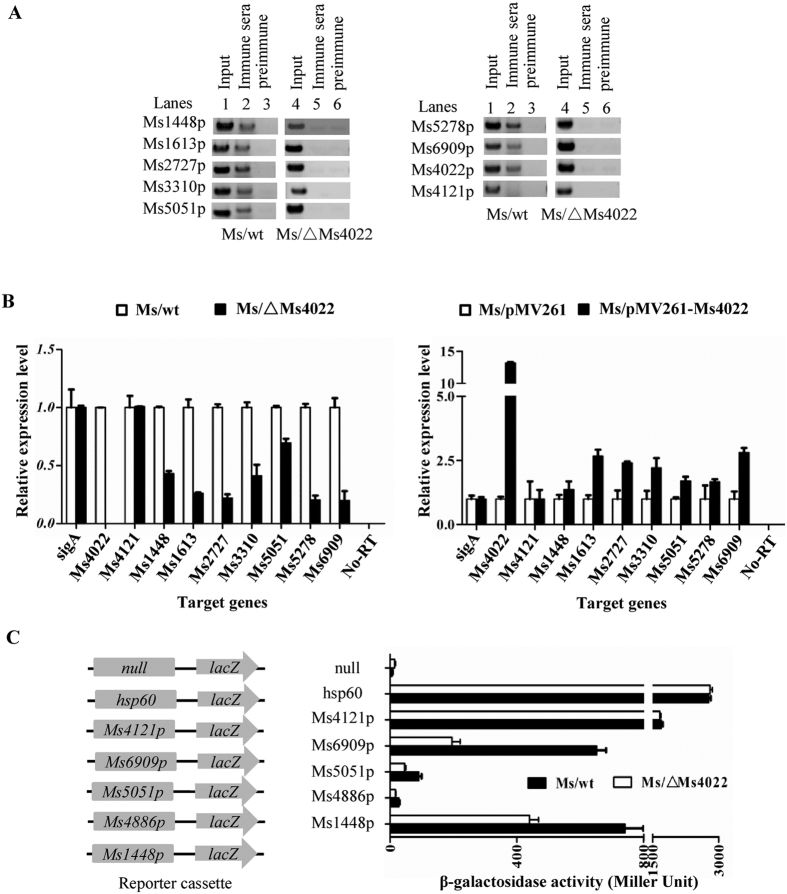
Interaction of Ms4022 with the promoter of its target genes *in vivo* and expression assays of target genes in different recombinant strains. (**A**) ChIP assays for the binding of Ms4022 to promoters of target genes. ChIP using pre-immune or immune sera rose against Ms4022. DNA recovered from the immunoprecipitates was amplified with primers specific for the target genes or an unrelated mycobacterial promoter of Ms4121. (**B**) Quantitative real-time PCR (qRT-PCR) assay for the regulation Ms4022 to its target genes in *M.smegmatis*. The relative expression levels of the genes were normalized using *sigA* rRNA gene as an invariant transcript, and an unrelated promoter gene Ms4121 was used as negative control. The cDNA of Ms/wt or Ms/pMV261was negative control. Data were analyzed using the 2^−ΔΔCt^ method. The P-values of the relative expression data were calculated by unpaired two-tailed Student’s T-test using GraphPad Prism5. (**C**) β-galactosidase activity assay. Left panel: Schematic representation of plasmids containing null-*lacZ* and promoter-*lacZ*. All of the plasmids were transformed into the Ms/wt or Ms/ΔMs4022.The activity of β-galactosidase was further examined and presented as Miller units. Null-*lacZ*, hsp60-*lacZ* and Ms4121p-*lacZ* were used as controls. Relative expression data were analyzed for statistical significance, P ≤ 0.05.

ChIP assays were established to detect the binding ability of Ms4022 *in vivo* for these promoters. As shown in Fig. 6A (see also Supplementary Fig. 5C), a specific Ms4022 antiserum recovered the target promoters by immunoprecipitation in wild-type *M. smegmatis* but not in Ms4022-deleted *M. smegmatis*. The Ms4022 antiserum could not recover the negative control Ms4121p.

In conclusion, Ms4022 can bind with the 22 promoters of target genes both *in vitro* and *in vivo*.

### Ms4022 positively regulates the expression of transport-related genes

The expressions of target genes were detected by quantitative real-time PCR (qRT-PCR) assays. As shown in Fig. 6B, the expression of seven transport-related genes was down-regulated in Ms/ΔMs4022 compared with Ms/wt. As expected, the expression levels of these genes were up-regulated in the Ms/pMV261-Ms4022. The expression of Ms4121 without the conserved motif did not change considerably. Meanwhile, no signal was detected in the no-reverse transcriptase control. Several other transport-related genes were also activated by Ms4022 (Supplementary Fig. 6A and B). These findings suggest that Ms4022 functions as a transcriptional activator and regulates the expression of transport-related genes.

Furthermore, the β-galactosidase experiment assay confirmed the regulation of Ms4022 to its target genes. Reporter plasmids were constructed and transformed into Ms/wt and Ms/ΔMs4022 (Fig. 6C). The expressions of *lacZ* connected with all four promoters (Ms1448p, Ms4886p, Ms5051p, and Ms6909p) were down-regulated in Ms/ΔMs4022 compared with Ms/wt. However, the expressions of *lacZ* connected with the strong promoter *hsp60* had no difference in Ms/wt and Ms/ΔMs4022. Similar results were obtained when the promoter was substituted by the negative control promoter Ms4121p. Again, *lacZ* connected with the null promoter exhibited no signal of galactosidase activity (Fig. 6C).

These results show that Ms4022 can function as a transcriptional activator and positively affect the expressions of transport-related genes.

### Seven transport-related genes overexpressing *M. smegmatis*is strains showed significant RIF resistance

**Figure 7:**
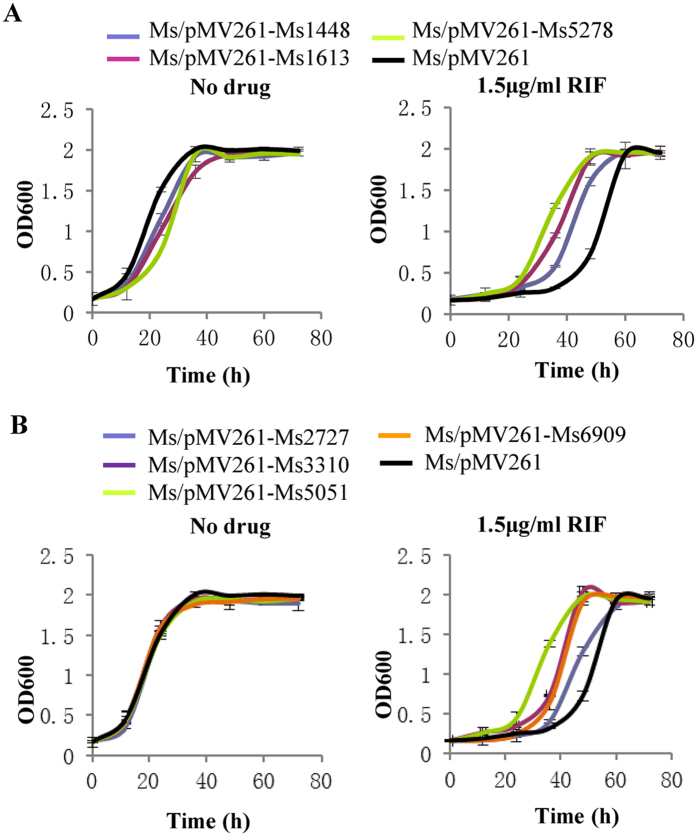
The growth curves of seven target genes overexpressed in *M. smegmatis* in response to RIF. All strains were grown in 7H9 medium that contains 30 μg/ml Kan without or with 1.5 μg/ml RIF. Ms1448, Ms1613 and Ms5278 overexpressing strains affected *M. smegmatis* growth. All of these seven target genes overexpressed strains showed resistance to RIF. Aliquots were taken at the indicated times and the OD600 was measured. Each analysis was performed in triplicate. (**A**) Growth curves of Ms1448, Ms1613 and Ms5278. (**B**) Growth curves of Ms2727, Ms3310, Ms5051 and Ms6909.

We transformed the overexpression plasmids of 14 transport-related genes in *M. smegmatis* and detected the growth and drug susceptibility of these recombinant strains. The growth curves of the recombinant strains showed that the Ms/pMV261-Ms1448, Ms/pMV261-Ms1613, and Ms/pMV261-Ms5278 strains grew much slower than the Ms/pMV261 strain in the 7H9 medium (Fig. 7A). The other recombinant strains did not affect the growth of *M. smegmatis.* The drug susceptibility of these strains was revealed by the growth curves in the presence of RIF. Seven transport-related genes overexpressing *M. smegmatis* strains (Ms/pMV261-Ms1613, Ms/pMV261-Ms1448, Ms/pMV261-Ms2727, Ms/pMV261-Ms3310, Ms/pMV261-Ms5051, Ms/pMV261-Ms5278, and Ms/pMV261-Ms6909) grow faster than the wild-type control strain when RIF was present in the 7H9 medium (Fig. 7A,B).

These results indicate that the seven transport-related genes activated by Ms4022 could result in RIF resistance of *M. smegmatis*.

## Discussion

A new TetR family transcriptional factor, Ms4022, was functionally characterized in *M. smegmatis*. We proved that the overexpression of Ms4022 caused RIF resistance in *M. smgmatis* through elevated levels of transport-related genes.

The genomes of both fast-growing *M. smegmatis* and pathogenic *M. tuberculosis* encode many putative drug transporters and membrane proteins^22^. Several regulators and transporters involved in mycobacterial multidrug resistance have been demonstrated^23^. For example, *M. tuberculosis iniBAC* is involved in the tolerance to INH and EMB^8^. A histone-like protein Lsr2 negatively regulate the expression of *iniBAC*^24^. In addition, LfrA was the first efflux pump described in *M. smegmatis,* belonging to the major facilitator family (MFS) protein. LfrA overexpression contributes to the resistance of fluoroquinolones. LfrR, a TetR family transcriptional repressor, inhibits the expression of LfrA^7^. The deletion of *lfrR* resulted in resistance to several drugs^25^. Usually, TetR family transcription factor binds a conserved palindromic sequence and represses the expression of target genes^10^. Unlike LfrR, Ms4022 acts as an activator and recognizes three19 bp non-palindromic sequence motifs (motif 1 from −39 to −57, motif 2 from −238 to−256 and motif 3 from −258 to −277) in its own promoter proved by EMSA and footprint assays. We got 315 potential target genes positively regulated by Ms4022. By analyzing the binding motif position of these genes, we found 95% (133/140, motif 1), 96% (130/136, motif 2) and 92% (68/74, motif 3) were outside the −35 region which is the RNA polymerase recognition site. The binding of Ms4022 to promoters may located upstream of ribosomal binding site, and thus enhance the transcription of target genes^26^. For this case, transcriptional factor usually functions as an activator. For example, Ms6564 is a TetR family activator broadly affects the expressions of diverse genes in *M. smegmatis*^15^. Compared with the conserved region in target genes’ motif, only 9 bp shown high similarity. Therefore, the binding pattern of Ms4022 may be affected and regulated by its long C-terminal non-conserved region (Supplementary Fig. 5A). Ms4022 binds to those target promoters may favor the RNA polymerase to promote transcription of target genes.

In this study, we identified a TetR famlily regulator Ms4022 and seven transport related genes contributing drug resistance to RIF, but not to INH and EMB in *M. smegmatis*. They are Ms1448 (sulfate permease), Ms1613 (polar amino acid transport system permease), Ms2727 (glutamate transport system substrate-binding protein), Ms3310 (integral membrane protein), Ms5051 (major facilitator superfamily protein), Ms5278 (transmembrane protein), and Ms6909 (amino acid ABC transporter permease). The possible mechanism would be a substrate preference to RIF. Transport related genes always show preference to a certain type of substrates. Based on the range of substrates, transporters can be divided into two categories. One is multi-substrates efflux system which shows broad substrate specificity. The other is single-substrate efflux system which tends to transport structural analogs. It is possible that 7 target genes of Ms4022 belong to single-drug efflux systems. Specific transport proteins have also been reported. For example, *E. coli* TetB can extrude tetracycline and a narrow range of close structural analogs^27^. In *Bacillus cereus*, BC4707 show a more specific role in the efflux of norfloxacin, and MmpL7 is a INH efflux in *M. smegmatis*^28^,^29^. Drug-specific response transcription factors also exist. Such as, FurA, OhrR and Ms0535 showed specific drug resistance^20^,^30^,^31^. In addition, Ms1448, Ms1613, and Ms5278 overexpressed strains could partially inhibit the growth of *M. smegmatis*. It appears that these target genes may be important in determining the RIF resistance of *M. smegmatis* only when their expression is increased. Interestingly, a homologous protein of Ms4022, MAV_1977, was also found in the pathogen *M. avium*. Six transport-related genes (except Ms3310) have their homologs in pathogen *M. avium* and *M. tuberculosis*. Therefore, our findings in *M. smegmatis* provided important clues for further understanding of the function of transport-related genes and their regulation of bacterial drug resistance in pathogenic mycobacteria. Unexpectedly, Ms4022 has no homolog in *M. tuberculosis*. The regulation of target genes remains to be identified in *M. tuberculosis*.

In sum, we presume that Ms4022 is a novel TetR family transcription factor that contributes to the RIF resistance of *M. smegmatis*. The findings of this study enhance our understanding of transport-related gene regulation and drug resistance in mycobacteria.

## Additional Information

**How to cite this article**: Liu, H. *et al.* Novel TetR family transcriptional factor regulates expression of multiple transport-related genes and affects rifampicin resistance in *Mycobacterium smegmatis. Sci. Rep.* **6**, 27489; doi: 10.1038/srep27489 (2016).

## Supplementary Material

Supplementary Information
