# Supplementary material for: Novel TetR family transcriptional factor regulates expression of multiple transport-related genes and affects rifampicin resistance in Mycobacterium smegmatis
Source: Sci Rep. 2016 Jun 7;6:27489. doi: 10.1038/srep27489 (PMC4895335; doi:10.1038/srep27489)
Supplement: Supplementary Information [file srep27489-s1.pdf]

Novel TetR family transcriptional factor regulates expression of multiple transport-related genes and affects rifampicin resistance in *Mycobacterium smegmatis*

Huicong Liu, Min Yang*, Zheng-Guo He*

National Key Laboratory of Agricultural Microbiology, College of Life Science and Technology, Huazhong Agricultural University, Wuhan 430070, China

*To whom correspondence should be addressed: College of Life Science and Technology, Huazhong Agricultural University, Wuhan 430070, China
Email: hezhengguo@mail.hzau.edu.cn or yangmin@mail.hzau.edu.cn

Tel: +86-27-87284300, Fax: +86-27-87280670

Supplemental figure 1

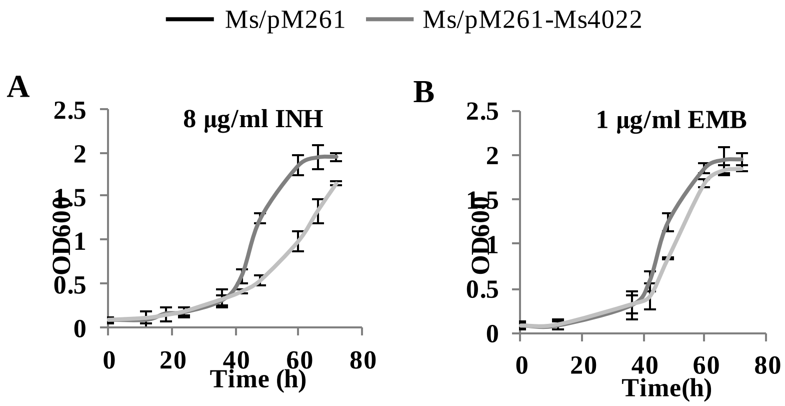

The effect of Ms4022 on *M. smegmatis* growth in response to isoniazid (INH) and Ethambutol (EMB). Ms/pMV261 . and Ms/pMV261-Ms4022 were grown in 7H9 medium which containing 30 µg/ml Kan and 8 µg/ml INH or 1 µg/ml EMB. Aliquots were taken at the indicated times and the OD600 was measured. Each analysis was performed in triplicate.

Supplemental figure 2

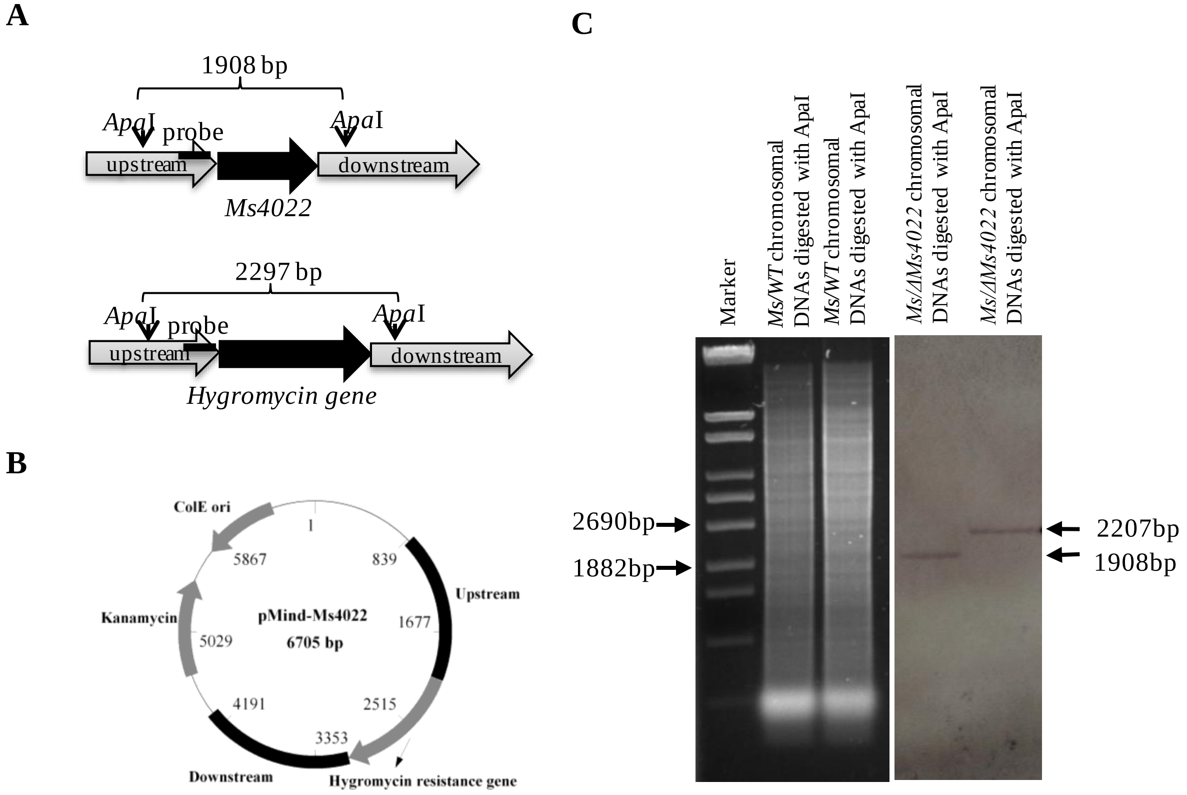

Construction of the Ms4022-deleted *M. smegmatis* strain and Southern blot assays. (A) Schematic representation of the DNA fragments of the Ms/wt and Ms/△Ms4022 knockout strain. The chromosomal DNAs were treated with the restriction enzyme *Apa*I. A 391bp probe was indicated with a black bar. (B) The recombinant plasmid of Ms4022 containing the upstream and downstream, and the hygromycin resistance gene. (C) Southern blot assays. A 391bp probe was used to detect the size change of the ApaI-digested genomic fragment of Ms/wt and Ms/△Ms4022. The probe corresponded with the sequence of the Ms4022 upstream genomic fragment.

Supplemental figure 3

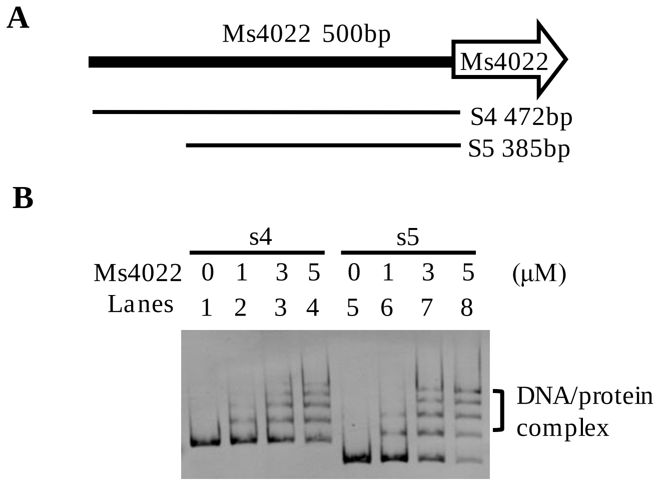

Binding ability of Ms4022with different truncated DNA fragments. (A) DNA fragments of different truncated and EMSA assay. The promoter of Ms4022 was truncated to different length to detect the DNA-binding region of Ms4022 on its own promoter. (B) EMSA assays. Different DNA fragments s4 and s5 were incubated with various amounts of Ms4022 protein. Ms4022 could bind both s4 and s5.

Supplemental figure 4

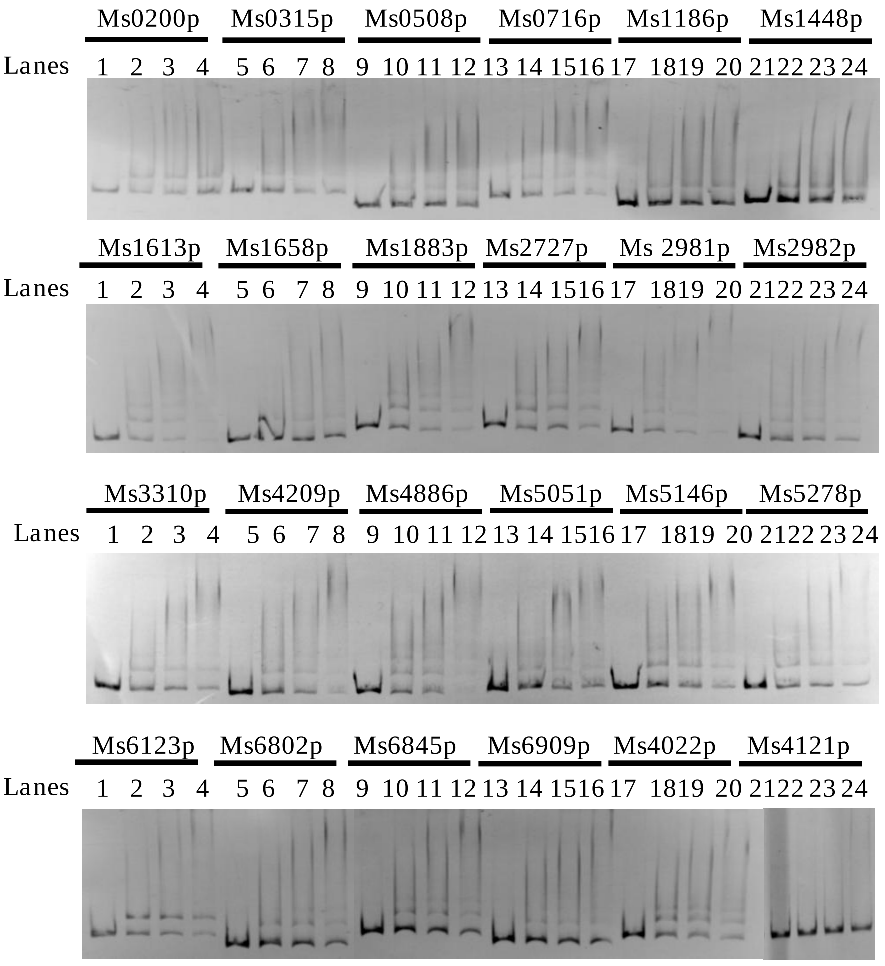

EMSA assay for specific binding of Ms4022 to its target genes. The promoter of Ms4022 target genes were co-incubated with various amount of Ms4022 protein. 22 promoters of ABC transporter or membrane protein were bound to Ms4022. Ms4121p was used as a negative control.

Supplemental figure 5

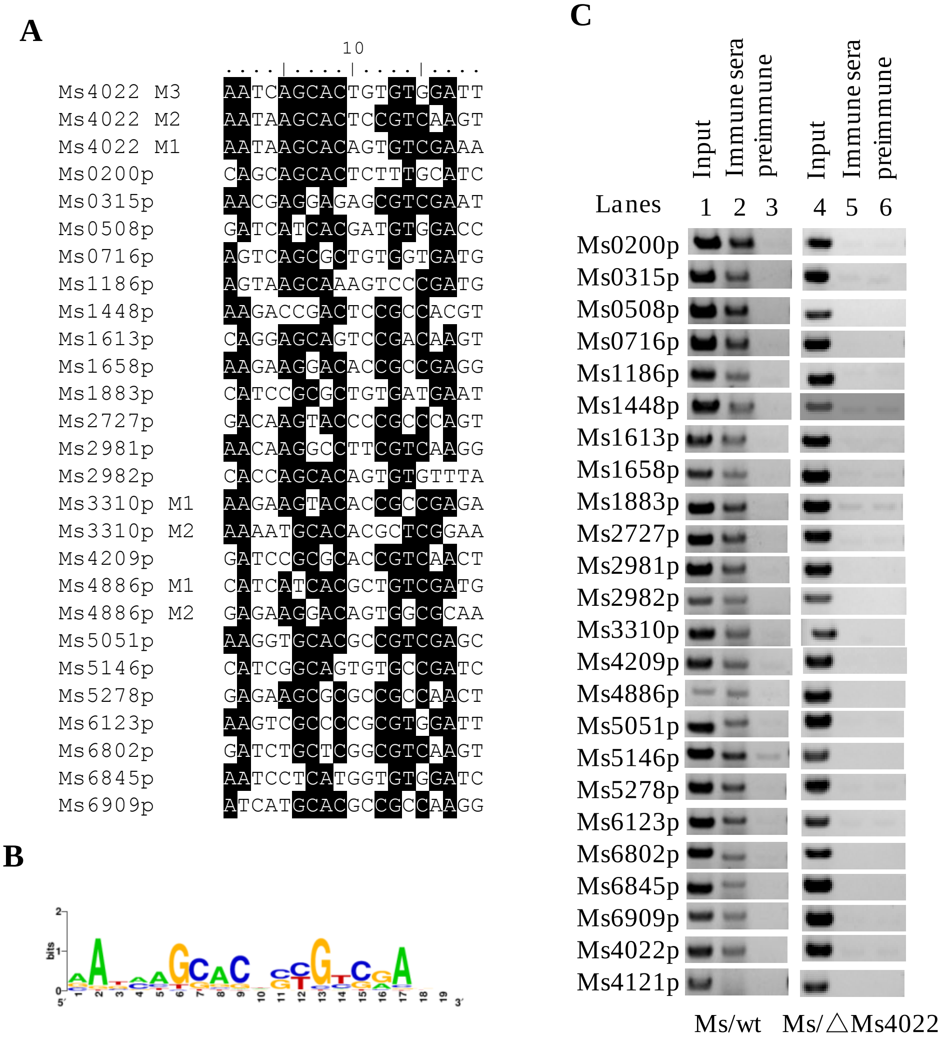

Interaction of Ms4022 with the promoter of its target genes in vivo. (A) Three binding motif sequence were used to search the promoters in the *M. smegmatis* genome. Promoters of 22 target genes were identified and listed. Conserved sequences are highlighted. (B) Logo assays for the protected region. The logo was generated by MEME software suite. (C) ChIP assays for the association of Ms4022 with target genes. ChIP using preimmune or immune sera rose against Ms4022. DNA recovered from the immunoprecipitates was amplified with primers specific for the target genes or an unrelated mycobacterial promoter of Ms4121.

Supplemental figure 6

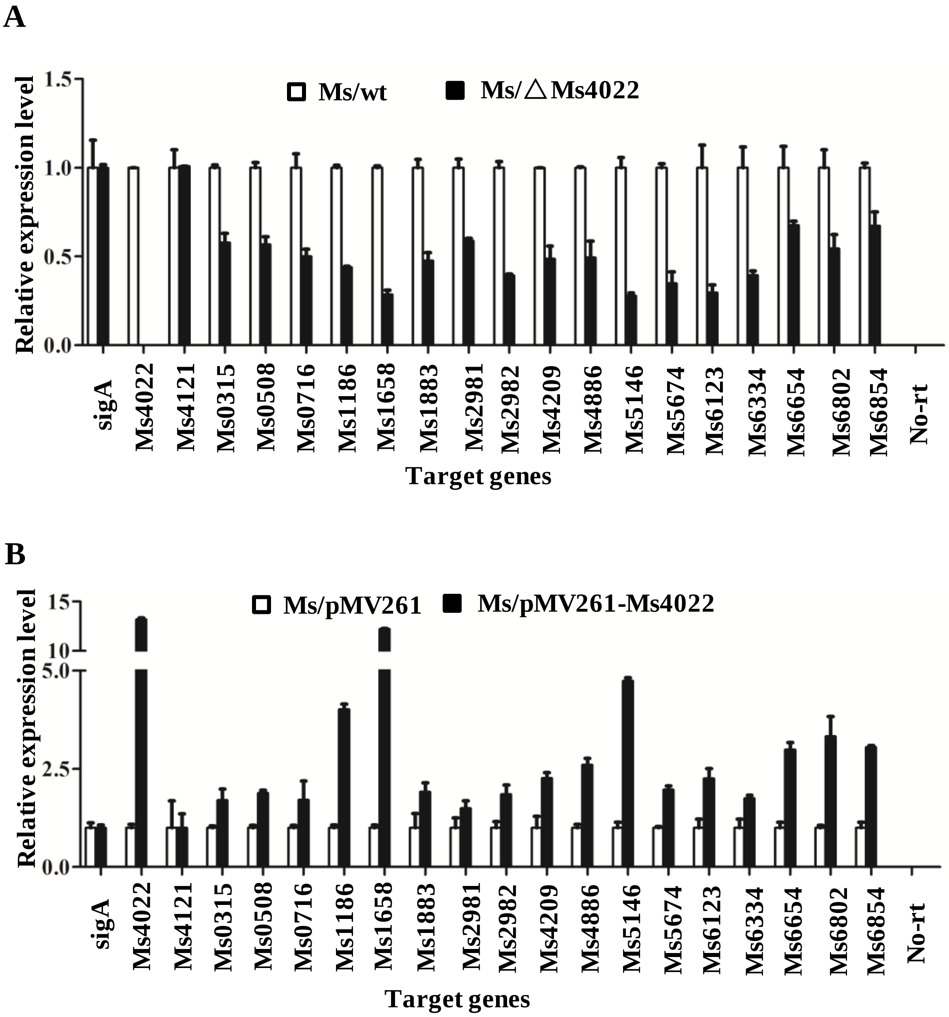

qRT-PCR assay for the regulation Ms4022 to the additional target genes in different recombination strains. Data were analyzed using the $2^{-\triangle\triangle Ct}$ method. The P-values of the relative expression data were calculated by unpaired two-tailed Student's t-test using GraphPad Prism5.

Supplemental figure 7

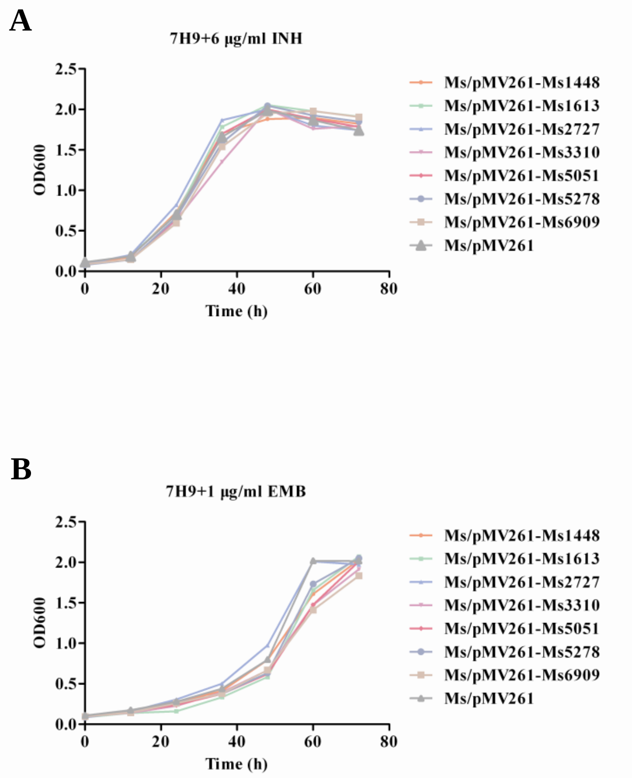

The growth curves of target genes overexpressed in *M. smegmatis* in response to INH and EMB. Seven target genes Ms1448, Ms1613, Ms2727, Ms3310, Ms5051, Ms5278 and Ms6909 were overexpressed in *M. smegmatis*. All strains were grown in 7H9 medium containing 30 μg /ml Kan and 6 μg /ml INH (A) or 1 μg /ml EMB (B). All of these seven target genes overexpressed strains did not shown resistance to INH and EMB. Aliquots were taken at the indicated times and the OD600 was measured.

Supplemental figure 8

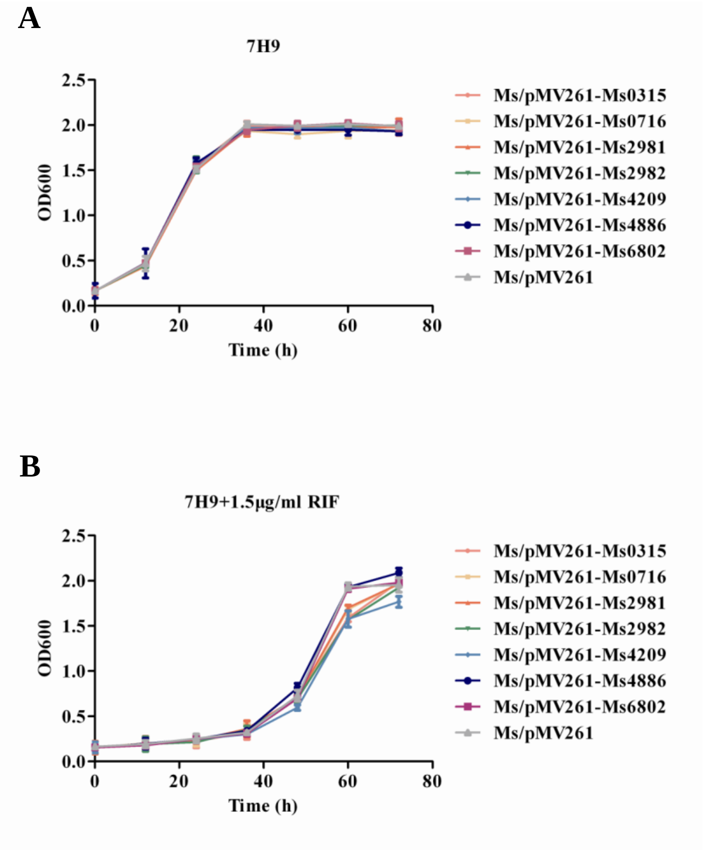

The growth curves of the additional target genes overexpressed in *M. smegmatis* in response to RIF. The additional target genes Ms0315, Ms0716, Ms2981, Ms2982, Ms4209, Ms4886 and Ms6802 were overexpressed in *M. smegmatis*. All strains were grown in 7H9 medium containing 30 μg /ml Kan and no drug (A) or 1.5 μg /ml RIF (B). Aliquots were taken at the indicated times and the OD600 was measured.

Supplemental Tables

Supplemental Table S1 Primers for DNA amplification and plasmid construction in this study

Name	Sequence 5'–3'	Restriction enzyme sites	Usage
Ms4022upF	GCGCGC**TTAATTAA**TGAATGCCGACATCCCTTCG	*PacI*	Knock out
Ms4022upR	ATATGC**ACTAGT**CGGGCGAACTTCCCTGTCAC	*SpeI*	Knock out
Ms4022dnF	ATATGC**AAGCTT**GCCGGTCGGTCAGGCATAGT	*HindIII*	Knock out
Ms4022dnR	ATATGC**GCTAGC**AGATCGAACCGTGGACGAAC	*NheI*	Knock out
HygF	AGCCAGCGCATATGGTGACACAAGAATCCCTG		Knock out detect
HygR	ACACTTAATTAATTAGGCGCCGGGGGCGGT		Knock out detect
Ms4022F	GACG**GAATTC**ACTTGACCCGACGGAGATCG	*EcoRI*	Clone and expression
Ms4022R	ATAT**TCTAGA**TCATGCGCTGCGCGCAGCCC	*XbaI*	Clone and expression
Ms4022pF	GACG**GAATTC**ACTTGACCCGACGGAGATCG	*EcoRI*	Clone and expression
Ms4022pR	ATAT**TCTAGA**TCATGCGCTGCGCGCAGCCC	*XbaI*	Clone and expression
Ms4022pR*	ATAT**TCTAGA**TCATGCGCTGCGCGCAGCCC		DNA Footprinting
Ms4121pF	GACG**GAATTC**AGATGGCTCTCGCAAGCAGT	*EcoRI*	Clone and expression
Ms4121pR	ATAT**TCTAGA**TCAGTTCGCCCGGTGCGCCT	*XbaI*	Clone and expression
Ms4022s1F	TGGATTTCGTCACGGATGGT		EMSA
Ms4022s1R	ACGCCGATACTTGACGGAGT		EMSA
Ms4022s2F	AACCGCCTCGGCGAATGCTC		EMSA
Ms4022s2R	GCCTTACGGCACAACGACTT		EMSA
Ms4022s3F	ACTCCGTCAAGTATCGGCGT		EMSA and southern blot
Ms4022s3R	GTCGATCTCCGTCGGGTCAA		EMSA
Ms0020pf	ATAT**GAATTC**CCGGGGTGCCGGTGATGGCG	*EcoRI*	EMSA
Ms0020pr	AGAC**TCTAGA**GGCGCGAAGCCAATGTCGGC	*XbaI*	EMSA

Ms0315pf	ATAT**<u>GAATTC</u>**GGCGAACCGGACGAGTCCAT	*EcoRI*	EMSA
Ms0315pr	ATAT**<u>TCTAGA</u>**CGAGCATGACCGCAGCAGGA	*XbaI*	EMSA
Ms0508pf	ATAT**<u>CTCGAG</u>**GCGTTCATCATCCCGCTGTA	XhoI	EMSA
Ms0508pr	AGAT**<u>TCTAGA</u>**GGGTTGTGCTCCTGTTGGTG	XbaI	EMSA
Ms0716pf	AGAT**<u>GAATTC</u>**TCGCTGGTGACCAGTCGCCC	*EcoRI*	EMSA
Ms0716pr	ATAT**<u>TCTAGA</u>**GTCGACCCCTTCCGTGGCGG	*XbaI*	EMSA
Ms1186pf	GACG**<u>GAATTC</u>**CGAACTCGCCACGTACGCAT	*EcoRI*	EMSA
Ms1186pr	ATAT**<u>TCTAGA</u>**GGATACCCCGAGGGCGGCCA	*XbaI*	EMSA
Ms1448pf	ATAT**<u>GAATTC</u>**CAAGATCGACCCGGCCACCA	*EcoRI*	EMSA
Ms1448pr	GCGC**<u>TCTAGA</u>**CAAAACTCCGTTCGTACGAA	*XbaI*	EMSA
Ms1613pf	ATCG**<u>GAATTC</u>**AGAAGGTCGTCGACTTCGTT	*EcoRI*	EMSA
Ms1613pr	ATCG**<u>TCTAGA</u>**TGTGCCGCACTCACTTCTGG	*XbaI*	EMSA
Ms1658pf	ATCG**<u>GAATTC</u>**GTGGTGCCCGAGCCGATGAT	*EcoRI*	EMSA
Ms1658pr	ATCG**<u>TCTAGA</u>**GCCACCTCCTGGCGTTACGA	*XbaI*	EMSA
Ms1883pf	ATAT**<u>CTCGAG</u>**TTCCTCGTCGACCTGCCGGT	XhoI	EMSA
Ms1883pr	GCGC**<u>TCTAGA</u>**GGTCAATTCATCACAGCGCG	XbaI	EMSA
Ms2981pf	ATAT**<u>GAATTC</u>**ACCATGCCGGTGGTCTCGGT	*EcoRI*	EMSA
Ms2981pr	AGAT**<u>TCTAGA</u>**ACGGTTGTCAGATCCCCCTC	*XbaI*	EMSA
Ms2982pf	ATATAT**<u>GAATTC</u>**ACCCATGGCGCGTCCGGAGC	*EcoRI*	EMSA
Ms2982pr	GCGCGC**<u>TCTAGA</u>**GAACAACCTTTCCCGATGAT	*XbaI*	EMSA
Ms3310pf	ATAT**<u>GAATTC</u>**CGGCGTGCCCGTCGATCACC	*EcoRI*	EMSA
Ms3310pr	AGACTCTAGACGTCATTCCTCCTGCTCGGG	*XbaI*	EMSA
Ms4209pf	ATATAT**<u>GCGGCCGC</u>**ATCTGTTCCCGATGGAGACC	*NotI*	EMSA
Ms4209pr	AATTCG**<u>TCTAGA</u>**AGCAACCCCAAATCGGTCAG	*XbaI*	EMSA
Ms4886pf	ATCG**<u>GAATTC</u>**AACCCGACGCCGACGTACTC	*EcoRI*	EMSA
Ms4886pr	ATCG**<u>TCTAGA</u>**CAGCGGCTTGGCCTCCTTGA	*XbaI*	EMSA
Ms5051pf	AGAT**<u>GAATTC</u>**TCGAGGAGCCGATGTACACC	*EcoRI*	EMSA

Ms5051pr	AGAT**TCTAGA**GCGCGGGCGATCCAAGAAAT	*XbaI*	EMSA
Ms5146pf	AGAT**GAATTC**GGTAAGGCCGCCATGATGCT	*EcoRI*	EMSA
Ms5146pr	AGATTCTAGACTGCTGGTGGCCTGGTTCAT	*XbaI*	EMSA
Ms5278pf	ATCG**GAATTC**CTCGCGATGATGTGTGACAT	*EcoRI*	EMSA
Ms5278pr	ATCG**TCTAGA**GCGTCGAACTTTAGCGATGC	*XbaI*	EMSA
Ms6123pf	ATCG**GAATTC**ATGGTGGTCGGCACCGTCGA	*EcoRI*	EMSA
Ms6123pr	ATCG**TCTAGA**GGTCACCCGAATGCGGTTGT	*XbaI*	EMSA
Ms6802pf	ATCG**GAATTC**ACATTCCTCGGGACGTCGTT	*EcoRI*	EMSA
Ms6802pr	ATCG**TCTAGA**CGTGATCTCCTCCGCGCGAA	*XbaI*	EMSA
Ms6845pf	ATATA**GAATTC**CACCGTCGCCGTGCGTCGAC	*EcoRI*	EMSA
Ms6845pr	GCGCG**TCTAGA**CGGAAGTGCCTTTCGAAGTT	*XbaI*	EMSA
Ms6909pf	ATATAT**GAATTC**CGCCCGCTTCGACGAGGCCA	*EcoRI*	EMSA
Ms6909pr	GCGCGC**TCTAGA**CTAAAGCAACCTAATGCGAG	*XbaI*	EMSA
Ms4022F1-F	CCGCCATCTCAATAAGCACAGTGTCGAAAAAAATAAGCA		synthesized directly
Ms4022F1-R	TGCTTATTTTTTTCGACACTGTGCTTATTGAGATGGCGG		synthesized directly
Ms4022F2-F	CCGCCATCTCCGATCGATCGATCGATCGAAAAATAAGCA		synthesized directly
Ms4022F2-R	TGCTTATTTTTCGATCGATCGATCGATCGGAGATGGCGG		synthesized directly
Ms4022F3-F	AGCTGATAAAAATCAGCACTGTGTGGATTCGTGCTAACC		synthesized directly
Ms4022F3-R	GGTTAGCACGAATCCACACAGTGCTGATTTTTATCAGCT		synthesized directly
Ms4022F4-F	AGCTGATAAACGATCGATCGATCGATCGACGTGCTAACC		synthesized directly
Ms4022F4-R	GGTTAGCACGTCGATCGATCGATCGATCGTTTATCAGCT		synthesized directly
Ms4022F5-F	AAAATAAGCACTCCGTCAAGTATCGGCGTGGACGCCTAGT		synthesized directly
Ms4022F5-R	ACTAGGCGTCCACGCCGATACTTGACGGAGTGCTTATTTT		synthesized directly
Ms4022F6-F	AAATCGAGAGTAATGCAGATCATCGGCGTGGACGCCTAGT		synthesized directly
Ms4022F6-R	ACTAGGCGTCCACGCCGATGATCTGCATTACTCTCGATTT		synthesized directly
Ms0315F	ATAT**GAATTC**AAGTGCCGGCCTATGCGCTGGT	*EcoRI*	Clone and expression

Ms0315R	ATATAT**<u>TCTAGA</u>**TCAGCCCGGACGCGGTCGAT	*XbaI*	Clone and expression
Ms0716F	ATCG**<u>GAATTC</u>**GCGTGACAACCATCGACAATGA	*EcoRI*	Clone and expression
Ms0716R	ATCGCG**<u>TCTAGA</u>**TCAGTAACTCACTTTCGCTG	*XbaI*	Clone and expression
Ms1448F	ATCG**<u>GAATTC</u>**ATGTGACCTCTGCAACGATCAC	*EcoRI*	Clone and expression
Ms1448R	ATCGAT**<u>TCTAGA</u>**TCAGTGTGCGCCGGTGAGCT	*XbaI*	Clone and expression
Ms1613F	ATCGA**<u>GAATTC</u>**GAATGAACTTCGACTGGACATTCTTC	*EcoRI*	Clone and expression
Ms1613R	ATATA**<u>TCTAGA</u>**TCACCTCACCCCCGCGGGTTCCAG	*XbaI*	Clone and expression
Ms2727F	ATCGA**<u>GAATTC</u>**GAATGCGGTCCATTCCCAAACGCGTC	*EcoRI*	Clone and expression
Ms2727R	ATATA**<u>TCTAGA</u>**TCAGTCGCGGTCGACGGCGGGAGG	*XbaI*	Clone and expression
Ms2981F	ATCGA**<u>GAATTC</u>**GAATGGATATCCTGATCGGCCAGCTG	*EcoRI*	Clone and expression
Ms2981R	ATATA**<u>TCTAGA</u>**TCATACCAGACTCCTTGTCCGCAC	*XbaI*	Clone and expression
Ms2982F	ATCGA**<u>GAATTC</u>**GAATGCGAGTTCGAGGACGTGCCACG	*EcoRI*	Clone and expression
Ms2982R	ATATA**<u>TCTAGA</u>**TCAGCTCGACAGCCCTGCGGCCCA	*XbaI*	Clone and expression
Ms3310F	ATAT**<u>GAATTC</u>**ATATGAGCGTGAGCGCCCGCCC	*EcoRI*	Clone and expression
Ms3310R	ATATAT**<u>TCTAGA</u>**TCACATCCGGCCGTGGCGGC	*XbaI*	Clone and expression
Ms4209F	ATAT**<u>GAATTC</u>**GCATGACCGAGGATTCGCTGCC	*EcoRI*	Clone and expression
Ms4209R	ATGCGC**<u>TCTAGA</u>**TCACAGAATCCCGTCCAGAA	*XbaI*	Clone and expression
Ms4886F	ATAT**<u>GAATTC</u>**ATATGGCCACGTCGACGGGGGT	*EcoRI*	Clone and expression
Ms4886R	ATATAT**<u>TCTAGA</u>**CTAGCCCTTCACCACCGCGG	*XbaI*	Clone and expression
Ms5051F	ATCG**<u>GAATTC</u>**ATATGACCGTAACCGCAGACCT	*EcoRI*	Clone and expression
Ms5051R	ATATGC**<u>TCTAGA</u>**TTAGTGCTCGGGGCGCAGCA	*XbaI*	Clone and expression
Ms5278F	ATCG**<u>GAATTC</u>**ATGTGACGGGCACCAAGACCAC	*EcoRI*	Clone and expression
Ms5278R	ATATGC**<u>TCTAGA</u>**TCAGGCGCTCTCGTTGTTGG	*XbaI*	Clone and expression
Ms6802F	ATCGA**<u>GAATTC</u>**GAGTGACCGGTACAGGGAGCACACCC	*EcoRI*	Clone and expression
Ms6802R	AGCAA**<u>TCTAGA</u>**TCAGAGACCGGCGCGTTCCTCATC	*XbaI*	Clone and expression
Ms6909F	ATCGA**<u>GAATTC</u>**GAATGAGCGTCAGACGACTCGCGGCG	*EcoRI*	Clone and expression
Ms6909R	ATATA**<u>TCTAGA</u>**TCAGATCATCTCCTGGGTGGCGGC	*XbaI*	Clone and expression

Supplemental Tables

Supplemental Table S1 Primers for qRT-PCR in this study

Name	Sequence 5'–3'
Ms0135F	GAGAAGGTCAACACGATCGC
Ms0135R	CTCGAAGTTCACCAGGGTCT
Ms0508F	CGAGTATCTCAACCGCAAGC
Ms0508R	CATGCGTGACGTAGATGGTG
Ms0716F	TTCTTCCCGAACCTGAGTCC
Ms0716R	CAACAACGACAGGACAAGCA
Ms1186F	GTGTTGTATGCCACCCTCAC
Ms1186R	TACGTATCGAGTACCACGCC
Ms1448F	CTGCTGGAATCGCTGATGAC
Ms1448R	GACGTTGATCATGGTCTGGC
Ms1613F	CGAAAGTCATGCGGTGGATC
Ms1613R	GTCAGCGCCAGGTAGTAGAT
Ms1658F	TCGATTCTGCTATGGGTGCT
Ms1658R	TGTAGGCGAACACCATGAGT
Ms1883F	GGATCCAGTGGTTGTCGAAC
Ms1883R	CAGGCCCAGTAGAAGATCGT
Ms2727F	TTGCAGCAGTACGACACCTA
Ms2727R	TCGCGTCGTTGATCTTGTTG
Ms2981F	ATCTTCATCCTGGTCCTGGC
Ms2981R	TCGAACCGATGAGTGTCAGG
Ms2982F	CAACATCTTCTACACCGGCG
Ms2982R	TGTAGTCCTCGCCCTTGATC
Ms3310F	AAGTTCTTCAAGGACCTGCG
Ms3310R	AGTTGTACAGTCCGAAAGCC
Ms4022F	CTCGAAGCAGGAGGTCATCA
Ms4022R	CTGATGGCGTCGATGAACAG

Ms4121F	CAGTTCCACACCCTCATTGC
Ms4121R	AAGTATCGCCGCATGTTGAC
Ms4209F	CTTGCTGGTCGGGTTGTTC
Ms4209R	CCGGAGTAGAACGCGTAGAA
Ms4886F	CGTCTTCCCCAAGCTGTTCT
Ms4886R	ACAAGGGAGTACACGAGCAT
Ms5051F	TTCTTCCCCGACCAGAGTTC
Ms5051R	CAGGACCAGCAGCAAAATCG
Ms5146F	CTACTTCATCCCGTTGCTGC
Ms5146R	GCCACGACACGATGAAGATC
Ms5278F	TCTGGTGTTCATCTGGCTGT
Ms5278R	GTCCTGCCACACGTGAAAAT
Ms5674F	TCTATGCGATCAACAGCCCA
Ms5674R	GGACAGATCGACCCAGTTGA
Ms6123F	ATCCCACCAACCTGTACCTG
Ms6123R	GAGGAGCATTGTGGTGACG
Ms6334F	ATCTTGTATCCCTCACCCGC
Ms6334R	TACTTGGACTTCAGCCCCAC
Ms6654F	GACCATCGGAACACTGTTGC
Ms6654R	CGCAGCAGTGAATCGATGTC
Ms6802F	GATCCTGATGAACGGTGAGC
Ms6802R	CCGACTTGATGCCGATCTTC
Ms6845F	ATCGTGCTGTTCATCATCGC
Ms6845R	GGATCGGCGTCATGATCAAG
Ms6909F	TCGACTGGGACCTGTACAAG
Ms6909R	AGCAGGATGATCACCACCTC
SigAF	GGGCTACAAGTTCTCGACCT
SigAR	ATCTCCTTGGCGAGCTCTTC

Supplemental Table S3 Strains and plasmids used in this study

Strain or plasmid	Relevant genotype or features	Source or reference
**Strains**		
E.coli		
DH5a	Host for plasmid construction	TaKaRa
BL21	Host for overexpression	TaKaRa
**M. smegmatis mc^{2}155**		
Ms/wt	M. smegmatis mc2155	This study
Ms/pMV261	M. smegmatis mc^{2}155 with pMV261	This study
Ms/pMV261-Ms4022	M. smegmatis mc^{2}155 with pMV261-Ms4022	This study
Ms/pMV261-Ms0315	M. smegmatis mc^{2}155 with pMV261-Ms1613	This study
Ms/pMV261-Ms0716	M. smegmatis mc^{2}155 with pMV261-Ms2727	This study
Ms/pMV261-Ms1448	M. smegmatis mc^{2}155 with pMV261-Ms6909	This study
Ms/pMV261-Ms1613	M. smegmatis mc^{2}155 with pMV261-Ms1613	This study
Ms/pMV261-Ms2727	M. smegmatis mc^{2}155 with pMV261-Ms2727	This study
Ms/pMV261-Ms2981	M. smegmatis mc^{2}155 with pMV261-Ms6909	This study
Ms/pMV261-Ms2982	M. smegmatis mc^{2}155 with pMV261-Ms1613	This study
Ms/pMV261-Ms3310	M. smegmatis mc^{2}155 with pMV261-Ms2727	This study
Ms/pMV261-Ms4209	M. smegmatis mc^{2}155 with pMV261-Ms6909	This study
Ms/pMV261-Ms4886	M. smegmatis mc^{2}155 with pMV261-Ms1613	This study
Ms/pMV261-Ms5051	M. smegmatis mc^{2}155 with pMV261-Ms2727	This study
Ms/pMV261-Ms5278	M. smegmatis mc^{2}155 with pMV261-Ms6909	This study

Ms/pMV261-Ms6802	M. smegmatis mc^{2}155 with pMV261-Ms2727	This study
Ms/pMV261-Ms6909	M. smegmatis mc^{2}155 with pMV261-Ms6909	This study
Ms/△Ms4022	M. smegmatis mc^{2}155 Ms4022 replaced by hyg	This study
Ms/pMindD	M. smegmatis mc2155 with pMindD	This study
△Ms4022/pMindD	Ms/△Ms4022with pMindD	This study
△Ms4022/pMindD-Ms4022	Ms/△Ms4022 with pMindD-Ms4022	This study
**Plasmids**		
pET28a	Kan^r, lacZ operon, T7 promotor, His-Tag	TaKaRa
pET-Ms4022	overexpression N His-tag Ms2173	This study
pMv261	Kan^r, pAL5000 replicon	This study
pMv261-Ms4022	Ms4022 in *EcoRI-XbaI* site of pMv261	This study
Ms/pMV261-Ms0315	Ms0315 in *EcoRI-XbaI* site of pMv261	This study
Ms/pMV261-Ms0716	Ms0716 in *EcoRI-XbaI* site of pMv261	This study
Ms/pMV261-Ms1448	Ms1448 in *EcoRI-XbaI* site of pMv261	This study
Ms/pMV261-Ms1613	Ms1613 in *EcoRI-XbaI* site of pMv261	This study
Ms/pMV261-Ms2727	Ms2727 in *EcoRI-XbaI* site of pMv261	This study
Ms/pMV261-Ms2981	Ms2981 in *EcoRI-XbaI* site of pMv261	This study
Ms/pMV261-Ms2982	Ms2982 in *EcoRI-XbaI* site of pMv261	This study
Ms/pMV261-Ms3310	Ms3310 in *EcoRI-XbaI* site of pMv261	This study
Ms/pMV261-Ms4209	Ms4209 in *EcoRI-XbaI* site of pMv261	This study
Ms/pMV261-Ms4886	Ms4886 in *EcoRI-XbaI* site of pMv261	This study
Ms/pMV261-Ms5051	Ms5051 in *EcoRI-XbaI* site of pMv261	This study
Ms/pMV261-Ms5278	Ms5278 in *EcoRI-XbaI* site of pMv261	This study
Ms/pMV261-Ms6802	Ms6802 in *EcoRI-XbaI* site of pMv261	This study
Ms/pMV261-Ms6909	Ms6909 in *EcoRI-XbaI* site of pMv261	This study

pMindD	Kan^r, pAL5000 replicon	This study
pMindD-Ms4022	Ms4022 in *EcoRI-XbaI* site of pMindD	This study
pMv261-LacZ	Kan^r, pAL5000 replicon	This study
pMv261-hsp60-LacZ	Kan^r, pAL5000 replicon	This study
pMv261-Ms4121p-LacZ	Ms4022 in *EcoRI-XbaI* site of pMv261-LacZ	This study
pMv261-Ms1448p-LacZ	Ms4121 in *EcoRI-XbaI* site of pMv261-LacZ	This study
pMv261-Ms4886p-LacZ	Ms1613 in *EcoRI-XbaI* site of pMv261-LacZ	This study
pMv261-Ms5051p-LacZ	Ms2727 in *EcoRI-XbaI* site of pMv261-LacZ	This study
pMV261-Ms6909p-LacZ	Ms6909 in *EcoRI-XbaI* site of pMv261-LacZ	This study
